# Retraction: Knowledge and attitude of the communities towards COVID-19 and associated factors among Gondar City residents, northwest Ethiopia: A community based cross-sectional study

**DOI:** 10.1371/journal.pone.0347174

**Published:** 2026-04-16

**Authors:** The PLOS One Editors

This article [1] was identified as one of a series of submissions for which we have concerns about authorship and adherence to the journal’s first and second publication criteria.

In addition, the article [1] reports the same study and data set as the previously published articles [2, retracted in 3] and [4, 5, retracted in 6] that were not cited or discussed in [1].

In light of these issues, the *PLOS One* Editors retract this article.

ZNA and MWM did not agree with the retraction. AAT, AGM, DMG, GMK, MKY, SYT, KAG, HSM, AWA, CAW, GMB, NTA, CDA, TA, ATT, BKR, EBT, ZA, HD, KTG, GGK, THM, SD, JA, TA, and MA either did not respond directly or could not be reached.
